# Supplementary material for: HS-SPME/GC×GC-TOFMS-Based Flavoromics and Antimicrobial Properties of the Aroma Components of Zanthoxylum motuoense
Source: Foods. 2023 May 31;12(11):2225. doi: 10.3390/foods12112225 (PMC10252244; doi:10.3390/foods12112225)
Supplement: Supplementary file 1 [file foods-12-02225-s001.zip › foods-2389525-supplementary.pdf]

Supporting information

Table S1.

Identification of volatile compounds in MEO and BEO.

NO.	compounds	CAS	rt	MEO-1	MEO-2	MEO-3	MEO-4	MEO(relative content %)	BEO-1	BEO-2	BEO-3	BEO-4	BEO(relative content %)
1	(+)-4-Carene	29050-33-7	28.5	0.147223799	10.28813041	7.007129632	117.6368689	0.084±0.049	189.177981	270.0662432	280.7112211	320.3275622	13.941±2.519
2	(3H)Indazole, 3,3-dimethyl-	59341-22-9	47.1667	7021.835941	5189.506946	5534.304819	1.5043769	37.02±3.376	20.93640468	23.74297927	33.59209292	23.2082248	0.278±0.040
3	Ethanol, 2-(ethenyloxy)-	764-48-7	47.1667	1184.953113	1329.644498	1238.696759	14.24650239	0.067±0.078	0.147223799	47.72009607	0.147223799	0.147223799	0.171±0.019
4	(3-Methyl-oxiran-2-yl)-methanol	92418-72-9	41.5	0.147223799	23.1294614	17.55964585	48.1068453	31.820±6.350	0.147223799	309.9401905	0.147223799	1254.084353	15.173±3.722
5	α-Phellandrene	99-83-2	16	4507.030823	4365.135458	6293.068021	0.147223799	5.525±0.393	10.73467916	13.81501973	17.23965961	13.30927889	25.567±0.193
6	α-Pinene	80-56-8	19.6667	0.147223799	792.5104271	0.147223799	66.39002594	0.975±0.005	165.2059228	183.474399	342.7158872	179.5138514	8.696±1.990
7	α-Terpineol	98-55-5	30.4167	166.7273503	133.027837	151.0989398	106.8549365	56.220±11.634	288.9672662	344.1283433	397.3053884	346.901256	67.807±19.995
8	β-Myrcene	123-35-3	16	8363.363771	7353.77643	11289.25187	18.5565441	7.618±0.444	24.03527571	15.84745423	23.30758609	30.62767254	0±0

9	β-Ocimene	13877-91-3	18.6667	1406.987418	1082.111405	1138.643461	1.721416388	86.215±3.214	7.814839999	7.368357394	10.49916787	9.605879132	48.478±16.236
10	β-Phellandrene	555-10-2	17.5833	14281.25636	8929.914589	13292.54585	4.653679664	5.590±0.271	8.994064067	9.948017696	9.377972826	11.12671637	35.583±3.335
11	β-Pinene	127-91-3	14	1006.557358	847.3084093	818.8755164	2.182732065	0.244±0.279	6.65434751	7.248536282	8.848149569	6.195099736	0.314±0.249
12	γ-Glutamyl-(S)-allyl-L-cysteine	91216-95-4	45.1667	35.79880714	0.147223799	19.18075315	10792.71505	6.859±3.837	12660.37914	18553.37838	15713.44023	13954.16135	38.933±8.181
13	γ-Terpinene	99-85-4	25.0833	1593.594621	507.7533004	590.8469883	11.59714017	0.001±0.000	26.50722609	34.53969842	36.45173891	23.83981754	0.071±0.042
14	1-(2-Methoxyethoxy)-2-methyl-2-propanol	211321-90-3	47	35.91060447	0.147223799	0.147223799	18.11435106	0.006±0.008	35.41220697	43.10423094	50.66953421	40.65138945	5.452±4.729
15	1,1,1-Tris(hydroxymethyl)propane	77-99-6	39.3333	2.666591405	0.147223799	0.147223799	12.9439333	0.001±0.000	1406.291746	1516.576335	1699.135934	1409.362498	12.845±1.330
16	1,11-Dodecadiyne	20521-44-2	31.25	15.52577835	0.147223799	810.917766	8.957362986	0.001±0.000	36.10521362	76.69207166	176.5851002	147.2328329	0.252±0.203
17	1,2-Propanediol	57-55-6	38.5833	0.147223799	4.701545597	0.147223799	1.435525938	0.720±0.471	2.838236237	2.306009696	5.654539554	5.095597131	0.3578±0.216
18	1,3,8-p-Menthatriene	18368-95-1	22.9167	79.7691598	65.54243218	194.8937868	36.01095086	0.010±0.001	4476.728175	5801.298366	5032.264162	9103.836556	0.075±0.11
19	1,5-Diacetoxypentane	6963-44-6	16.8333	1.863633532	1.339249182	1.811193216	0.147223799	0.570±0.185	1072.053836	1220.667204	1451.426446	1184.116093	0±0
20	1,6-Octadiene, 3,7-dimethyl-	2436-90-0	24.6667	142.631315	59.92477562	86.39970932	27.33164896	9.075±0.720	10.79736504	18.61382131	24.10189941	0.147223799	5.280±0.815
21	1,7-Octadien-3-ol, 2,6-dimethyl-	22460-59-9	27.25	1641.708592	1245.442496	58.80776225	0.147223799	0.001±0.000	0.147223799	50.60372044	225.995166	0.147223799	11.092±1.549

				0.1472	0.1472	9.7534	10.909		232.46	432.99	447.24	421.83	
22	1,8-Nonadiyne	2396-65-8	19.25	23799	23799	57573	57956	0.117±0.010	0827	77538	7048	78121	0.092±0.032
				21.169	13.416	16.269	120.99		116.34	135.90	170.75	126.67	
23	1-Butanamine, 3-methyl-	107-85-7	37.3333	39644	48782	67135	89928	0.544±0.269	9696	5267	86464	73125	0.145±0.127
				42.946	117.20	93.418	35.587		91.374	108.19	120.84	105.78	
24	1-Butanol, 3-methoxy-	2517-43-3	45.9167	29397	96838	88362	31104	0.045±0.014	25567	48441	51473	78046	0.055±0.012
				11.114	4.7952	6.2109	739.55		9233.6	5.4828	10776.	3647.7	
25	1-Butanol, 3-methyl-	123-51-3	17.3333	28485	88206	38768	19496	0.001±0.000	56557	9039	06932	80092	0.020±0.010
				0.1472	0.1472	0.1472	12.141		293.64	335.35	383.17	331.89	
26	1-Butene, 3-methyl-	563-45-1	28.6667	23799	23799	23799	77927	0.176±0.015	17536	47082	48571	22791	3.569±0.529
	1H-Cyclopenta[1,3]cyclopropa[1,2]benzene, octahydro-7-methyl-3-	13744-15-		26.454	27.214	28.210	0.1472		49.892	36.904	44.089	56.751	
27	methylene-4-(1-methylethyl)-, (3aS,3bR,4S,7R,7aR)-	5	26.8333	78862	88997	52686	23799	0.690±0.097	15123	75149	9961	23839	0.492±0.176
				127.65	87.280	1119.4	2.9305		42.032	42.930	6.3276	45.606	
28	1-Heptanol	111-70-6	26.0833	51562	43332	72554	55051	0.145±0.020	55326	11707	9501	6657	0.111±0.023
				28.309	21.759	23.982	10.692		5.2421	6.8488	10.329	4.2242	
29	1-Hexanol	111-27-3	21.75	54163	79971	32225	68243	0.263±0.019	84186	81222	86725	83354	1.111±0.023
				48.822	37.501	37.796	13.947		6.7466	13.240	11.475	13.908	
30	1-Octanol	111-87-5	27.25	22302	70191	50618	8477	0.001±0.000	45363	34592	68171	50707	0.056±0.007
				9.9974	7.0778	0.1472	391.71		17.198	0.1472	20.379	14.386	
31	1-Pentanol	71-41-0	18.6667	06399	96548	23799	92175	0.020±0.004	61985	23799	64652	90751	0.046±0.005
				2.8300	2.6569	3.6848	3.7078		10.169	15.146	14.194	14.552	
32	1-Penten-3-ol	616-25-1	15.6667	29615	63494	08408	44919	0.006±0.005	26981	70055	69352	30163	0±0
				2.0733	21.937	0.6203	302.38		9.4266	0.1472	0.1472	0.1472	
33	1-Propanol, 2-chloro-	78-89-7	11.75	59973	74805	96051	82031	0.238±0.045	83204	23799	23799	23799	0.366±0.017
				52.197	30.205	34.800	0.8306		0.9627	2.6193	1.9720	1.1431	
34	1-Propanol, 2-methyl-	78-83-1	13.5	1886	07261	77711	24452	0.296±0.082	9086	99588	82429	01774	0±0

35	1-Propen-2-ol, acetate	108-22-5	5.75	68.89483168	30.44679054	43.18152478	1852.6225	3.914±1.252	0.147223799	1879.742812	1995.711416	0.147223799	0±0
36	1-Propene, 2-methyl-	115-11-7	45.6667	862.0326044	385.1403858	0.147223799	5029.91458	0.053±0.016	2812.023029	5212.690767	6058.959616	3857.578254	0±0
37	2(3H)-Furanone, 5-ethyldihydro-	695-06-7	30.6667	9.284774444	5.674833832	11.42436863	8716.738841	0.507±0.160	31301.96853	18026.89136	16557.19937	27992.31248	1.958±0.618
38	2,4,6-Octatriene, 2,6-dimethyl-, (E,Z)-	7216-56-0	22.75	65.27863204	103.6785338	70.34593389	0.147223799	0.050±0.004	112.3277846	123.6273357	125.606643	128.5602609	0.649±0.157
39	2,4-Heptadienal, (E,E)-	4313-03-5	25.5	8.614294584	7.160124593	8.618677678	0.147223799	0.985±0.158	10.93522453	14.20049835	15.19658559	11.76068996	11.220±3.359
40	2,4-Hexadienal, (E,E)-	142-83-6	23	145.8823182	128.474926	176.1119894	33.99676367	1.187±0.164	10.80880181	11.43262313	11.6932094	10.52458655	0.046±0.049
41	2,6-Octadien-1-ol, 2,7-dimethyl-	22410-74-8	27.25	188.6393839	164.3485917	212.3812124	3.965264951	0.001±0.000	23.71634428	6.113968352	6.469509574	48.54819602	2.459±0.761
42	2,6-Octadien-1-ol, 3,7-dimethyl-, (Z)-	106-25-2	33.75	91.25618352	0.147223799	0.147223799	14.69747381	0.192±0.238	1.634222189	0.147223799	3.876039907	0.147223799	0±0
43	2-Butanol, (R)-	14898-79-4	37.75	12.7997604	5.45232149	71.76940347	1.619302488	0.080±0.092	4.771112763	4.08877289	8.368576894	75.26615396	4.228±0.302
44	2-Butenal	4170-30-3	11.6667	0.147223799	21.7326394	26.90329789	11.04932187	0.081±0.093	21.93239309	23.86106603	37.54603809	21.1403891	0.061±0.015
45	2-Butenal, (Z)-	15798-64-8	11.6667	26.14470005	0.147223799	0.147223799	0.147223799	0.077±0.006	30050.28786	37735.11617	43986.11389	27676.94027	0.089±0.020
46	2-Butenal, 3-methyl-	107-86-8	16.9167	13.40181153	11.4253034	12.99141832	907.1431141	0.226±0.007	42.91021743	34.79728741	42.77074968	34.41484818	34.540±7.498
47	2-Butene, 1-bromo-3-methyl-	870-63-3	20.25	38.52658903	35.4181373	34.07580274	6.888560045	0.008±0.005	12.92262664	15.73072479	23.34114269	13.13495662	0.006±0.002

48	2-Butenoic acid, ethyl ester, (E)-	623-70-1	15.8333	1.881455414	0.357193178	1.973366082	14337.15039	0.123±0.018	16082.18799	9835.080153	0.147223799	14094.98764	1.006±0.002
49	2-Caren-10-al	6909-19-9	32.75	20.1284465	15.07607918	21.48510865	19.18906189	0.525±0.063	49.65983018	61.41929124	62.93447046	63.03145323	2.006±0.002
50	2-Cyclohexen-1-one, 4-(1-methylethyl)-	500-02-7	30.0833	92.87560254	66.2391951	90.94953527	61.77981111	0.089±0.043	216.5098664	127.7087187	380.7229491	128.3744809	1.015±0.029
51	2-Furanmethanol, 5-ethenyltetrahydro-α,α,5-trimethyl-, cis-	5989-33-3	27.0833	17.89651436	5.604779581	11.66612235	3.215923543	1.23±0.198	1.219778249	0.147223799	0.147223799	0.147223799	52.449±17.576
52	2-Heptanone, 6-methyl-5-methylene-	498-51-1	28.3333	0.147223799	219.3982243	165.2691584	14.74701234	0.001±0.000	0.753157894	1.04325052	1.18965725	1.117879729	0.045±0.005
53	2-Heptanone, 7,7,7-trichloro-	154264-40-1	16.4167	37.07717738	27.29953519	0.147223799	38.68019492	0.031±0.005	7.047772515	0.147223799	0.147223799	4.491309666	0.029±0.002
54	2-Heptenal, (E)-	18829-55-5	20.8333	4.85351265	3.971513498	4.800708762	0.147223799	0.135±0.014	15.59308232	0.147223799	16.6998031	16.5505721	0.203±0.008
55	2-Hexenal	505-57-7	17.6667	25.87950072	21.50296342	19.97813893	1241.492873	0.126±0.038	0.147223799	30.52422076	4103.457097	0.147223799	0.572±0.067
56	2-Isopropenyl-5-methylhex-4-enal	75697-98-2	30.1667	21.94548767	11.16921424	21.25387013	0.813399849	0.001±0.000	0.147223799	4.922242207	0.147223799	0.147223799	1.875±0.575
57	2-Isopropylmalic acid	3237-44-3	38.5	8.111754023	3.268905397	0.147223799	370.278193	0.438±0.039	34.17202799	39.58198795	38.97969733	34.6367129	0.063±0.008
58	2-Nonen-4-yn-1-ol, (Z)-	134225-90-4	27.0833	78.64613797	59.34671818	71.42990631	337.4844943	0.516±0.054	895.4643055	1137.41634	1308.854414	1138.764624	0.316±0.197
59	2-Octanone	111-13-7	28	83.00179362	83.75855912	70.69809707	7.315758671	0.001±0.000	2555.99639	3706.962655	3218.998278	667.3773232	0.015±0.009
60	2-Octenal, (E)-	2548-87-0	23.75	22.16464207	22.28599767	0.147223799	23.31404589	0.018±0.012	0.147223799	14.6627409	3.477239142	14.18319672	0±0

61	2-Pentanol	6032-29-7	32.3333	1.5342<br>02441	1.0232<br>44739	3.7435<br>50068	930.24<br>24455	0.394±0.570	953.39<br>00791	7336.2<br>42621	2443.8<br>50665	8004.9<br>79829	0.605±0.363
62	2-Pentanone, 4-mercapto-4-methyl-	19872-52-<br>7	21	15.573<br>16144	0.1472<br>23799	190.61<br>41056	2.4444<br>7011	0.176±0.045	25.994<br>30226	16.221<br>92348	21.673<br>93628	47.429<br>86988	0±0
63	2-Pentanone, 5-bromo-	3884-71-7	19.6667	19.684<br>60258	30.095<br>33267	26.753<br>98223	35.021<br>46148	0.057±0.027	10.390<br>46319	0.1472<br>23799	0.1472<br>23799	17.523<br>66687	0.060±0.007
64	2-Penten-1-ol, (Z)-	1576-95-0	24.0833	3.5778<br>18728	8.4780<br>14699	10.423<br>22721	403.69<br>45872	0.025±0.006	66.617<br>7214	81.139<br>78004	88.871<br>35927	68.959<br>67546	0.156±0.015
65	2-Pentenal, (E)-	1576-87-0	14.6667	5.0157<br>34349	4.0498<br>30107	0.1472<br>23799	89.284<br>93982	0.132±0.061	33.799<br>00371	41.091<br>7792	47.467<br>15564	36.458<br>87546	0.423±0.031
66	2-Pentenal, 2-ethyl-	3491-57-4	16	32.322<br>38458	21.367<br>16189	10.234<br>86197	31.004<br>99722	0.001±0.000	0.8986<br>83108	0.1472<br>23799	0.9237<br>33864	0.1472<br>23799	0.724±0.548
67	2-Propanol, 1-(1-methylethoxy)-	3944-36-3	43.5	0.1472<br>23799	0.1472<br>23799	9.0778<br>56789	99.845<br>28226	0.001±0.000	7733.5<br>45188	12412.<br>56128	6174.5<br>0164	8973.6<br>79494	0.410±0.301
68	2-Propanol, 1-bromo-	19686-73-<br>8	46.75	0.1472<br>23799	71.049<br>42587	0.1472<br>23799	6.1294<br>91244	0.101±0.088	7.6970<br>72143	8.1599<br>76039	8.8670<br>35694	8.3879<br>29637	0.001±0.000
69	2-Propanol, 1-propoxy-	1569-01-3	47.25	32.315<br>32881	1.8975<br>29704	16.015<br>53839	52.190<br>49974	0.001±0.000	0.1472<br>23799	13.956<br>55795	0.1472<br>23799	0.1472<br>23799	0.047±0.005
70	2-Propanone, 1-hydroxy-	116-09-6	5.91667	36.936<br>54875	0.2944<br>47598	0.1472<br>23799	40.164<br>87978	0.001±0.000	196.23<br>77837	237.55<br>58725	60.462<br>32728	26.737<br>59989	0.983±0.983
71	2-Propenal	107-02-8	44.5	0.1472<br>23799	1262.6<br>70524	0.1472<br>23799	18.818<br>2347	0.376±0.012	29.759<br>72081	33.468<br>92448	41.071<br>75724	24.002<br>68202	0±0
72	2-Propyl-1-pentanol	58175-57-<br>8	25.6667	67.147<br>31409	54.608<br>7391	58.438<br>63138	0.1472<br>23799	0.041±0.010	128.15<br>88136	15.672<br>36007	177.88<br>95076	28.217<br>59081	0±0
73	2-Undecanone	112-12-9	32.8333	7.4091<br>58282	8.2119<br>9313	4.9039<br>48783	0.1472<br>23799	0.027±0.010	11.366<br>05087	13.572<br>05962	22.873<br>03659	14.744<br>55462	0±0

74	3,5-Octadien-2-one, (E,E)-	30086-02-3	26.1667	2.781977443	4.513445098	5.578593641	760.1429898	32.914±15.423	116.5825116	280.5156244	160.1669871	139.9757288	0±0
75	3,6-Nonadien-1-ol, (E,Z)-	56805-23-3	25.4167	5176.115285	6203.871978	1938.835832	945.5480475	0.095±0.058	15.20923948	16.28790427	29.64843394	29.26025081	0.036±0.022
76	3-Hexanone, 2,5-dimethyl-4-nitro-	59906-54-6	19.5	7.935481626	6.578331508	22.24626483	0.147223799	5.464±0.614	311.6047827	607.4454531	807.2314298	252.4127788	0.076±0.019
77	3-Hexen-1-ol, (Z)-	928-96-1	22.6667	0.147223799	3.839974995	775.7915439	209.4570093	5.231±0.372	44.1949071	29.27224473	0.147223799	11.14427508	0.181±0.017
78	3-Hexen-1-ol, acetate, (E)-	3681-82-1	26.0833	880.1561805	851.6126585	832.2520773	0.147223799	0.001±0.000	129.2450598	122.8896328	0.147223799	36.2177408	0.151±0.051
79	3-Hexen-1-ol, acetate, (Z)-	3681-71-8	26.6667	0.147223799	0.147223799	148.4921293	176.1814963	0.016±0.018	3870.362319	2466.456725	3693.09912	2815.344497	0.073±0.070
80	3-Hydroxy-2-butanone	513-86-0	35.25	4.30492716	0.147223799	5.748127138	772.85171	0.216±0.046	0.147223799	0.147223799	12.46501113	0.147223799	0.126±0.119
81	3-Isopropyl-4-methyl-1-pentyn-3-ol	5333-87-9	35.8333	29.54606391	27.77677428	41.33314604	38.01522085	0.470±0.885	228.8597889	190.3637736	222.875077	210.811731	0.161±0.180
82	3-Methyl-3-nitrobut-1-ene	1809-67-2	31.1667	7.502768778	0.147223799	277.1324931	1.140809659	0.001±0.000	30.19196902	46.59027876	3.411727896	86.03388399	0.305±0.269
83	3-Nonanone	925-78-0	21.75	0.147223799	14.22369386	0.147223799	0.147223799	0.001±0.000	2.351338792	8.358069217	3.817105079	3.561367654	0.060±0.014
84	3-Nonyne	20184-89-8	30.75	22.057285	0.147223799	1.051757712	20.03093216	0.582±0.650	16.80946045	17.10410547	21.05330786	19.85532962	0.774±0.181
85	3-Penten-1-ol, 4-methyl-	763-89-3	41.1667	15.87874892	65.25716107	41.50224937	5.687203297	0.001±0.000	6.361318829	6.617835354	8.828420313	4.098991542	0.163±0.037
86	3-Penten-2-one, (E)-	3102-33-8	14.5833	3.259214582	0.147223799	2.558488459	32.70586746	0.200±0.026	74.63471409	88.90462541	111.0284937	89.9381673	0.106±0.080

87	3-Phenyl-1-propanol, acetate	122-72-5	35.6667	0.1472<br>23799	26.259<br>40096	31.028<br>11909	281.26<br>95111	1.550±0.209	3721.6<br>06124	5921.6<br>89929	5519.7<br>395	4915.1<br>83098	9.748±0.463
88	4-Carene, (1S,3S,6R)-(-)-	5208-50-4	25	275.93<br>62509	268.88<br>24993	196.81<br>27129	13.614<br>93361	0.339±0.022	14.633<br>38248	18.549<br>42406	21.002<br>88865	15.779<br>17928	0±0
89	4-Hexen-1-ol, (E)-	928-92-7	20.9167	63.761<br>21744	48.728<br>57687	52.270<br>94827	79.041<br>23589	0.001±0.000	0.1472<br>23799	24.497<br>9503	41.227<br>23052	0.1472<br>23799	0.074±0.066
90	4-Penten-1-ol	821-09-0	16.5	17.275<br>42483	0.1472<br>23799	0.1472<br>23799	0.1472<br>23799	0.114±0.018	219.16<br>5547	367.88<br>39908	365.48<br>90971	384.87<br>3975	0±0
91	5-Hepten-1-ol, 2,6-dimethyl-	4234-93-9	43	23.350<br>37503	15.842<br>45687	15.856<br>37669	0.1472<br>23799	0.001±0.000	11.024<br>06044	7685.9<br>00079	0.1472<br>23799	5.5489<br>98733	0.025±0.004
92	5-Octen-2-yn-4-ol	609306-<br>99-2	28.5833	17.109<br>18865	0.1472<br>23799	29.235<br>51864	246.94<br>01181	0.082±0.015	2256.6<br>11336	2819.6<br>88456	3307.4<br>93854	640.26<br>00534	0±0
93	5-Tetradecene, (E)-	41446-66-<br>6	28.5833	11.512<br>50962	14.097<br>5171	15.019<br>27219	1759.9<br>28987	54.239±34.005	4173.1<br>04962	8806.5<br>2231	12780.<br>31617	14596.<br>41588	0±0
94	6-Octen-1-ol, 3,7-dimethyl-, acetate	150-84-5	29.4167	953.76<br>64509	10289.<br>34889	9164.0<br>85961	28394.<br>01877	146.851±4.667	881.53<br>37591	1081.5<br>41622	0.1472<br>23799	843.88<br>90816	0±0
95	6-Octenal, 3,7-dimethyl-, (R)-	2385-77-5	25.3333	32609.<br>66254	26299.<br>68356	18761.<br>81265	16361.<br>28529	0.048±0.021	0.1472<br>23799	0.1472<br>23799	697.72<br>94241	0.1472<br>23799	0.6060±0.293
96	7,8-Dihydro-α-ionone	31499-72-<br>6	36.9167	11.756<br>48084	8.0847<br>61601	2.9943<br>42973	5601.1<br>57484	5.914±1.876	2849.7<br>4058	1651.6<br>12917	0.1472<br>23799	6173.6<br>1725	5.0300±1.507
97	Acetaldehyde	59-66-5	47.1667	19.314<br>66168	25.272<br>66442	27.410<br>51229	7681.2<br>34616	0.125±0.044	0.1472<br>23799	15.559<br>62718	0.1472<br>23799	18.875<br>68753	0.304±0.128
98	Acetaldehyde, tetramer	108-62-3	44.8333	28.081<br>44588	16.860<br>35305	10.522<br>64352	5428.4<br>99581	0.333±0.362	7.2975<br>88739	0.1472<br>23799	4.8594<br>67416	9.9971<br>55638	0.399±0.263
99	Acetic acid	64-19-7	24.0833	333.19<br>01095	256.15<br>39847	285.75<br>04539	7.5236<br>40175	1.810±0.106	101.69<br>65928	221.52<br>33705	224.36<br>05044	223.74<br>53576	17.155±2.362

				103.48	80.760	106.10	14.003		170.52	0.1472	0.1472	176.65	
100	Acetic acid, 2-phenylethyl ester	103-45-7	33	40623	77335	89462	64972	0.628±0.075	70516	23799	23799	59482	2.531±0.262
				756.60	634.53	695.62	1100.7		109.43	352.09	182.13	231.52	
101	Acetic acid, heptyl ester	112-06-1	22.1667	64437	55856	11271	9292	4.463±0.226	84847	86717	18034	95206	0.599±0.250
				75.760	61.763	78.566	59.520		0.1472	1801.8	0.1472	0.1472	
102	Acetic acid, hexyl ester	142-92-7	17.9167	20969	32615	03817	92068	0.436±0.054	23799	12488	23799	23799	0.730±0.368
				28.340	2.6221	25.888	99.285		198.09	792.45	575.48	234.43	
103	Acetic acid, hydroxy-, ethyl ester	623-50-7	41.3333	07605	12163	8213	98089	0.090±0.088	36939	777	37525	74215	0.017±0.003
				5.9195	3.9753	0.1472	120.18		0.1472	195.19	105.49	147.91	
104	Acetic acid, octyl ester	112-14-1	29.9167	40957	37036	23799	90569	0.046±0.052	23799	76392	04748	51673	0±0
				17.107	11.491	13.980	765.20		46.887	48.601	0.1472	0.1472	
105	Acetic acid, pentyl ester	628-63-7	14.5	66801	27042	56287	94431	0.085±0.012	80358	44859	23799	23799	1.154±0.062
				28.689	18.848	18.689	0.1472		36.364	0.1472	0.1472	97.312	
106	Acetonitrile	75-05-8	10.4167	82287	72474	69391	23799	0.131±0.024	17926	23799	23799	15584	0.145±0.011
				0.1472	3.4894	0.1472	47.175		35.761	50.036	71.875	47.137	
107	Acetophenone	98-86-2	29.4167	23799	83996	23799	34067	0.001±0.000	73949	13485	36439	84473	0.492±0.055
				9.7656	5.2094	66.649	12.474		25.386	36.265	46.220	0.1472	
108	Allyl acetate	591-87-7	33.1667	0029	60893	84555	97806	0.1471±0.190	4223	68043	50276	23799	0.561±0.363
		14289-96-		7736.7	4742.2	4319.5	3404.1		38315.	32294.	66718.	30706.	
109	Allyl methallyl ether	4	25.4167	90458	93131	11709	88549	34.561±7.346	71949	25003	43452	24479	0.026±0.009
				0.1472	0.1472	0.1472	4.7905		44.758	4223.3	2423.3	35.753	
110	Aromandendrene	489-39-4	29.5833	23799	23799	23799	74925	0.001±0.000	58548	29057	89741	1046	1.181±0.116
				0.1472	0.1472	0.1472	1.8023		28.301	39.592	39.971	70.650	
111	Azulene	275-51-4	31.5833	23799	23799	23799	67277	0.001±0.000	68675	09244	13458	22209	0.010±0.004
				63.929	53.808	51.129	29.858		739.31	1052.1	1384.4	1034.1	
112	Benzaldehyde	100-52-7	26.25	70417	5209	04673	82564	0.338±0.034	32162	0944	10718	79145	0.173±0.034

113	Benzaldehyde, 4-(1-methylethyl)-	122-03-2	32.4167	8.623175112	9.428054064	8.473520297	833.4161664	0.064±0.018	21262.91937	22293.03071	22324.01053	28427.84713	0.551±0.034
114	Benzene, 1-methyl-3-(1-methylethyl)-	535-77-3	19.25	289.8011881	256.9407546	313.5069636	22.25935925	1.881±0.219	245.2123966	293.76796	341.3111473	305.933661	3.828±0.189
115	Benzene, 1-methyl-4-(1-methylethenyl)-	1195-32-0	24	32.18637868	30.88658533	38.17361903	0.147223799	0.211±0.026	15.99089658	20.26077719	17.60979394	0.147223799	0.311±0.022
116	Benzeneacetic acid, methyl ester	101-41-7	31.75	0.147223799	359.0198628	505.5140447	44.10086765	3.129±0.689	31.41663108	22.91049024	71.50447125	12.26446125	0.575±0.059
117	Benzoic acid, 2-methylpropyl ester	120-50-3	32.5833	28.88282229	28.59004525	28.90032315	1059.989781	0.187±0.014	24.94793023	5.966850046	21332.57008	4.60425086	0±0
118	Benzoic acid, methyl ester	93-58-3	28.6667	34.24149099	43.27476112	45.35281536	1512.572584	0.261±0.043	1165.796184	1666.948662	1429.362984	1913.024493	0.405±0.060
119	Benzyl alcohol	100-51-6	34.4167	32.15085657	23.65838418	23.16725184	133.6372487	0.165±0.016	35.1710456	38.36658413	55.98024511	18.75151174	0±0
120	Bicyclo[2.2.1]heptane-2-methanol	5240-72-2	15.8333	2651.283382	860.1386652	0.147223799	7389.860479	10.888±4.893	170.8186585	0.147223799	0.147223799	0.147223799	0±0
121	Bicyclo[3.1.0]hexan-2-ol, 2-methyl-5-(1-methylethyl)-, (1α,2α,5α)-	17699-16-0	24.6667	43.44749637	162.8474355	55.3849732	91.41357809	0.563±0.449	104.3831218	108.9983769	140.4632783	7.105027795	0.032±0.019
122	Butanal, 2-methyl-	96-17-3	7.83333	2.055538092	1.529239475	2.024125759	187.526436	0.011±0.001	2624.349224	5016.583951	3743.825143	3040.749748	0.030±0.003
123	Butanal, 3-methyl-	590-86-3	7.91667	5.380626727	3.243546988	3.45887527	0.147223799	0.026±0.005	3426.781189	4111.864582	5100.743987	4601.397867	0.034±0.004
124	Butane, 2-bromo-2-methyl-	507-36-8	33.5	0.147223799	0.147223799	0.147223799	187.2744253	0.001±0.000	19.19949881	11545.27021	1903.645604	20205.07688	0.120±0.115
125	Butanoic acid	107-92-6	28.6667	7.961150002	5.126749412	5.955610902	1.928823385	0.042±0.006	708.4739258	457.9387934	1065.372214	3158.149663	0.108±0.047

				368.65	285.54	316.16	22.245		3549.8	4428.7	3314.5	4860.4	
126	Butanoic acid, 2-methylpropyl ester	539-90-2	15.75	74175	30176	66368	36129	1.992±0.126	93542	88406	34332	46499	0±0
				9.1074	6.5567	7.7140	11.334		0.1472	2.1776	0.1472	0.5396	
127	Butyrolactone	96-48-0	28.9167	68013	08741	84792	83271	0.045±0.008	23799	67637	23799	92245	0.028±0.004
				14.761	11.313	11.808	0.1472		5.8713	8.7709	8.4329	6.5735	
128	Camphene	79-92-5	12.5833	32304	05838	60313	23799	0.076±0.006	30364	20434	07758	04814	0.141±0.008
				1379.3	1200.0	0.1472	42.451		102.06	119.88	150.38	100.11	
129	Caryophyllene	87-44-5	28.3333	08698	17764	23799	48142	7.982±0.036	80097	59611	75998	44813	1.492±0.075
				15.898	39.934	0.1472	7.6210		20.494	21.968	57.746	28.790	
130	CHF2CH2OH	359-13-7	42.25	82135	21932	23799	56456	0.174±0.086	51248	88783	60368	70412	0.079±0.018
		157477-		0.1472	8.8111	0.1472	7.1553		3.1454	4.4481	10.087	0.8327	
131	cis-Muurola-4(15),5-diene	72-0	40.1667	23799	89905	23799	75315	0.001±0.000	21789	60818	97309	73897	0.044±0.022
				27157.	18395.	32889.	5.5966	167.513±38.15	7.8591	8.6391	8.1694	7.6439	
132	Citronellal	106-23-0	25.3333	88648	82093	86264	67367	4	65223	09671	29451	90907	3.360±0.567
				2936.6	2171.2	65.106	0.1472		106.72	140.99	153.40	177.04	
133	Citronellol	106-22-9	32	60668	75261	88521	23799	15.979±1.418	82785	70232	71033	32814	0±0
				105.41	95.133	105.50	13444.		713.75	0.1472	0.1472	0.1472	
134	Copaene	3856-25-5	25.75	94091	98073	80033	01843	0.637±0.032	87042	23799	23799	23799	1.522±0.941
				2.5402	1.6899	0.1472	11.587		277.91	222.68	425.35	339.12	
135	Cycloheptylamine	5452-35-7	11.0833	56771	15976	23799	50767	0.009±0.006	83539	31242	38977	18547	0.013±0.004
				3.9872	0.1472	0.9508	19.493		305.85	0.1472	0.1472	362.51	
136	Cyclooctylamine	5452-37-9	32.3333	96222	23799	11913	60047	0.010±0.009	53003	23799	23799	43715	0±0
				0.1472	37.407	55.455	85.566		399.74	455.82	488.24	542.96	
137	Cyclopentane, nitro-	2562-38-1	33.75	23799	56377	85512	56085	0.354±0.104	38534	91425	3345	47123	0±0
				1665.8	1683.1	1425.3	254.37		1830.1	2468.4	3174.2	2591.1	
138	Decanal	112-31-2	25.6667	98359	70552	67574	93909	9.234±1.775	41845	94061	09243	66793	0.585±0.148

139	Diethylene glycol	111-46-6	44.75	80.055<br>58724	0.1472<br>23799	62.402<br>61801	26.162<br>74849	0.218±0.252	133.82<br>89186	154.91<br>50484	217.82<br>21954	165.11<br>42957	0.216±0.039
140	D-Limonene	5989-27-5	25.3333	0.1472<br>23799	9528.8<br>63011	3393.9<br>5675	156.51<br>62192	40.013±21.006	2458.8<br>67768	0.1472<br>23799	0.1472<br>23799	2270.0<br>02886	12.419±7.076
141	Dodecanoic acid, ethyl ester	106-33-2	41.4167	4.1043<br>24549	2.4660<br>06328	8.3460<br>47549	7.1562<br>29427	0.032±0.016	0.1472<br>23799	0.1472<br>23799	26.377<br>00297	0.1472<br>23799	0.015±0.006
142	Estran-3-one, 17-(acetyloxy)-2-methyl-, (2α,5α,17β)-	54550-10-<br>6	27.5833	62.136<br>20248	11.351<br>24982	0.1472<br>23799	0.1472<br>23799	0.423±0.383	11.710<br>94687	15.013<br>00462	25.771<br>71953	19.052<br>83706	0.150±0.020
143	Ethanamine, N,N-difluoro-	758-18-9	43.6667	588.17<br>83507	711.73<br>058	308.49<br>51864	234.94<br>04536	2.987±1.354	3723.5<br>42338	3275.3<br>09325	4307.7<br>41482	9643.1<br>93474	20.049±13.63<br>3
144	Ethanol	64-17-5	8.58333	11639.<br>25465	9410.2<br>27103	10363.<br>66546	6.0389<br>07863	66.122±2.553	19.137<br>49295	5.3988<br>87421	137.56<br>83895	38.447<br>02734	5.240±0.847
145	Ethanol, 2-(vinyloxy)-	75-07-0	47.1667	1390.4<br>90743	706.01<br>09184	625.50<br>90127	0.1472<br>23799	8.225±1.012	3354.8<br>93403	0.1472<br>23799	3861.0<br>61776	9063.1<br>72072	1.216±0.061
146	Ethanol, 2-[2-(ethenyloxy)ethoxy]-	929-37-3	44.5833	694.43<br>03397	252.19<br>01622	418.95<br>80893	3.3831<br>39799	2.877±0.978	1.5078<br>61298	3.2967<br>01805	4.7054<br>27459	2.7149<br>03915	3.015±0.450
147	Ethylmalonic acid	601-75-2	19.6667	2.8192<br>63495	1.6243<br>88443	10.845<br>60316	598.12<br>78992	0.028±0.028	119.94<br>3872	182.50<br>88173	191.12<br>37067	181.75<br>11733	0.010±0.004
148	Formaldehyde	50-00-0	47.0833	114.72<br>57766	126.32<br>46033	286.67<br>58859	2643.2<br>30166	1.309±0.647	0.1472<br>23799	0.1472<br>23799	0.1472<br>23799	82.178<br>92876	0.193±0.018
149	Formic acid	64-18-6	25.6667	23.305<br>85074	12.176<br>60668	16.510<br>27795	169.89<br>66989	0.103±0.025	0.1472<br>23799	10.430<br>243	12.799<br>64966	20.589<br>67058	0±0
150	Furan, 2-pentyl-	3777-69-3	18	4.8397<br>05254	3.9814<br>01959	6.5952<br>68864	134.92<br>68163	0.029±0.010	120.98<br>21456	0.1472<br>23799	0.1472<br>23799	140.18<br>24901	0±0
151	Furan, 3-(4-methyl-3-pentenyl)-	539-52-6	23.4167	66.514<br>42441	50.004<br>49595	34.183<br>91799	1.4760<br>01383	0.294±0.078	0.1472<br>23799	6.4355<br>02158	0.1472<br>23799	1.7798<br>28212	0.741±0.133

152	Furfural	98-01-1	23.5833	1.518509453	0.507563706	1.458169701	4.898240557	0.007±0.003	0.147223799	4.549959748	0.147223799	10.77078049	0.187±0.079
153	Germacrene D	23986-74-5	30.9167	238.6455759	205.300004	204.2210925	14.46031523	1.384±0.060	134.1843241	168.1715313	183.5555137	157.1064823	13.312±1.973
154	Heptanal	111-71-7	16.5	46.00186467	30.64337314	0.147223799	32.36170755	0.227±0.035	96.24199372	0.147223799	0.147223799	0.147223799	0.038±0.005
155	Heptanal, 2-methyl-	16630-91-4	17.1667	1496.563175	1115.541962	1257.484913	21.69308263	8.079±0.660	14.19557043	16.68514631	14.30143385	12.1312829	0.135±0.029
156	Heptanoic acid, 2-methyl-, methyl ester	51209-78-0	19.75	81.0991101	64.04294598	70.60021024	0.147223799	0.478±0.057	58.94309006	76.53884542	125.010381	100.4545697	0.136±0.013
157	Hexanal	66-25-1	13.0833	176.6381411	125.8412114	118.5919074	5.754678188	0.846±0.125	0.147223799	0.147223799	0.147223799	119.0920986	0.471±0.049
158	Hexane, 1-(ethenyloxy)-	5363-64-4	22.9167	242.3773441	180.9367264	28.62974826	110.5163272	1.305±0.106	539.3432733	749.674168	922.3564421	767.315027	0.142±0.057
159	Hexanoic acid, 5-methyl-, methyl ester	2177-83-5	18.3333	49.43899394	37.83294338	39.05526969	10.44864349	0.248±0.020	58.31678762	1390.172603	158.8563406	297.1412114	0±0
160	Hexanoic acid, ethyl ester	123-66-0	18.0833	16.85068013	11.92368183	13.30391056	7.108304223	0.089±0.008	45.30940521	6.20423007	80.79324086	7.532835346	0.003±0.000
161	Hexanoic acid, methyl ester	106-70-7	16.5833	29.42325715	10.97693862	0.147223799	2.902321886	0.112±0.052	183.8895304	374.3093885	512.969414	349.4913145	0.006±0.006
162	Humulene	6753-98-6	30.0833	234.1281244	210.1236831	250.0275904	34.63896141	1.490±0.133	643.497845	564.5301911	1120.317432	554.0945535	8.546±0.939
163	Hydroxylamine, O-(3-methylbutyl)-	19411-65-5	41.5	8.256457969	0.147223799	0.147223799	73.02728709	0.028±0.032	9.468721074	0.147223799	3.843579379	0.147223799	0.614±0.486
164	Isobutyl acetate	110-19-0	10.8333	22.33489274	12.01267797	15.69200519	26.33855332	0.098±0.024	10.97138946	0.147223799	13.4939677	0.147223799	5.200±0.515

165	Isoprene	78-79-5	23.9167	0.1472<br>23799	5.2509<br>92428	0.1472<br>23799	0.1472<br>23799	0.018±0.020	36.531<br>58889	15.095<br>12243	0.1472<br>23799	4.7651<br>06498	0.022±0.006
166	Isopulegol	89-79-2	27.75	6731.9<br>28795	4310.1<br>46958	5924.2<br>6661	42.681<br>3326	38.046±7.226	33.047<br>08223	43.773<br>31384	56.472<br>04318	48.450<br>6559	0±0
167	Lavandulol	58461-27-<br>1	26.5833	3474.8<br>44262	0.1472<br>23799	9.3267<br>32154	36.073<br>77558	0.030±0.030	0.1472<br>23799	0.1472<br>23799	52.606<br>62672	15.439<br>83489	0.149±0.049
168	Limonene	138-86-3	17.0833	7140.3<br>44032	6825.6<br>48066	5843.3<br>20698	38.667<br>33578	41.528±3.653	9.7671<br>8995	75.626<br>57619	24.306<br>86446	0.1472<br>23799	129.967±6.11<br>3
169	Limonene oxide, trans-	4959-35-7	24.6667	41.345<br>18344	0.1472<br>23799	33.818<br>75421	51.410<br>55264	0.115±0.132	10.598<br>05608	8.7121<br>78681	0.1472<br>23799	0.1472<br>23799	1.213±0.077
170	Linalool	78-70-6	26.5833	3251.0<br>68218	2772.1<br>46658	2853.1<br>16312	8.2060<br>28649	19.247±1.347	145.97<br>29792	271.93<br>37484	212.03<br>61275	71.313<br>13779	158.365±46.8<br>88
171	Linalool oxide	60047-17-<br>8	37.9167	3.0514<br>95384	0.1472<br>23799	1.5087<br>38192	19.746<br>74045	0.011±0.008	30.965<br>96039	30.730<br>68407	27.222<br>15707	16.007<br>14491	0.037±0.034
172	Linalyl acetate	115-95-7	26.9167	831.03<br>29927	654.15<br>53466	769.11<br>73155	8.6813<br>42282	4.837±0.370	0.1472<br>23799	46.403<br>34576	0.1472<br>23799	0.1472<br>23799	81.189±10.13<br>4
173	Methacrolein	78-85-3	7	27.896<br>77956	15.710<br>30308	31.367<br>61625	0.1472<br>23799	0.157±0.050	0.1472<br>23799	6.4419<br>87127	24.091<br>02303	0.1472<br>23799	0.630±0.051
174	Methyl Alcohol	67-56-1	7.5	107.24<br>14988	84.662<br>54023	84.091<br>57791	2.5781<br>86174	0.599±0.056	5.8496<br>93226	3.0580<br>39868	28.510<br>96647	8.2819<br>82167	1.180±0.047
175	Methyl salicylate	119-36-8	32.3333	91.069<br>08418	78.650<br>66371	111.82<br>08438	0.1472<br>23799	0.654±0.157	506.07<br>85832	669.00<br>65842	4.1705<br>45419	381.73<br>24539	0±0
176	Methylene chloride	75-09-2	8.25	3.9258<br>62433	1.1914<br>05706	4.4886<br>94477	1.8169<br>34641	0.018±0.009	7.8314<br>07865	84.503<br>2921	0.1472<br>23799	134.33<br>77461	0.025±0.004
177	α-Cadinene	24406-05-<br>1	32.75	0.1472<br>23799	3.5819<br>08142	0.1472<br>23799	1526.0<br>96128	0.001±0.000	513.11<br>85046	2537.4<br>11559	0.9943<br>03894	9832.5<br>18329	0.006±0.002

178	Nerolidol	142-50-7	37.4167	9.444867249	5.000089216	6.238756218	0.147223799	0.001±0.000	57.19511834	199.7993279	87.39630232	111.9404212	0.304±0.064
179	N-Nitroso(2-hydroxyethyl)glycine	80556-89-4	45.75	7.106916204	226.064629	154.3360402	8.276065878	0.046±0.011	0.147223799	45.06065557	34.97535824	61.12953246	0±0
180	Norpseudoephedrine	36393-56-3	3.75	140.0538939	158.2844294	83.02254709	76.23794361	0.741±0.640	207.8223697	92.16309606	0.147223799	195.5117705	0.489±0.266
181	Octanal	124-13-0	19.75	455.3373919	287.7977624	103.7677398	26.36166181	0.784±0.211	2.135523787	0.147223799	0.147223799	2.150569955	0.902±0.107
182	Octanoic acid, 2-methyl-, methyl ester	2177-86-8	14.9167	30.55783584	14.15298038	30.8753621	246.1074532	2.357±0.396	179.1688631	298.270866	199.2891735	221.7384566	0.263±0.028
183	Oxirane, 2,3-dimethyl-, cis-	1758-33-4	41.0833	32.1654547	0.147223799	25.33772989	1.685686013	0.140±0.058	4.643577284	4.334598455	1500.850845	0.147223799	0.038±0.009
184	Paraldehyde	123-63-7	46.6667	5.203198645	45.32314919	12.47990225	5.3878843	0.172±0.013	5.260235742	0.147223799	3.662221991	3.806668783	0±0
185	Penicillamine disulfide	20902-45-8	44.75	26.86061659	0.147223799	0.147223799	0.147223799	0.214±0.194	0.147223799	45.34637124	59.81775395	0.502699912	0.299±0.063
186	Pentanal	110-62-3	9.75	14.90809505	8.631879149	11.23700711	10.0780061	0.001±0.000	296.790761	56.78103035	257.0236361	76.48715391	0.157±0.060
187	Pentanoic acid, 3-methyl-, methyl ester	2177-78-8	14.75	18.10295622	10.45201517	12.42087381	26.31734286	0.072±0.012	49.71256297	11.39853934	213.5314196	284.4445529	0.114±0.006
188	Pentanoic acid, 4-methyl-, methyl ester	2412-80-8	15.1667	419.9815772	283.9325609	318.713941	1087.998219	0.080±0.018	0.147223799	15.48505004	0.147223799	0.147223799	0.023±0.008
189	Pentanoic acid, 4-oxo-	123-76-2	20.0833	0.147223799	18.26148202	24.71699927	35.12903221	2.208±0.282	16.09283841	0.435096169	67.08427486	5.664543972	0.060±0.012
190	Phenylethyl Alcohol	60-12-8	35.25	33.63487795	27.78820095	31.32502974	208.1563383	0.158±0.036	0.147223799	55.82706126	417.4438996	215.1790881	0±0

		21195-59-		18.844	11.491	26.663	291.39		2533.1	744.99	9629.0	0.1472	
191	p-Mentha-1,5,8-triene	5	23.75	41948	05068	50383	64864	0.212±0.037	63645	94103	48091	23799	0.154±0.016
				58.902	87.988	147.24	0.1472		115.97	1624.1	5.6170	1320.3	
192	p-Mentha-1,5-dien-8-ol	1686-20-0	32.25	9604	05158	45979	23799	0.121±0.040	92682	39196	20243	06879	0.064±0.004
				2.4627	2.0525	2.1152	494.23		704.73	132.50	1139.0	820.99	
193	Propanal	123-38-6	5.41667	65041	36821	73314	45505	0.735±0.332	34827	09599	44711	22263	0.047±0.016
				15.436	12.181	12.150	0.1472		53.375	50.467	82.065	18.696	
194	Propanal, 2-methyl-	78-84-2	5.66667	12151	48499	44231	23799	0.013±0.002	88514	08366	81578	60357	0.087±0.012
				154.07	0.1472	0.1472	11.968		33.978	27.090	76.756	49.333	
195	Propane	74-98-6	43.0833	83764	23799	23799	96383	0.085±0.006	28287	05269	24732	87388	0±0
				13.621	10.908	10.679	24.978		76.297	136.08	85.621	51.457	
196	Propanoic acid	79-09-4	26.4167	02675	15888	98896	56889	0.001±0.000	50967	73739	09088	06416	0.073±0.093
				0.1472	7.3346	0.1472	3.4278		37.854	29.028	14.956	0.1472	
197	Propanoic acid, 2-methylpropyl ester	540-42-1	22	23799	88887	23799	85807	0.075±0.005	85449	0027	96788	23799	0.124±0.019
				0.1472	0.1472	0.1472	30.808		51.224	136.35	74.762	126.33	
198	Propanoic acid, 2-oxo-, ethyl ester	617-35-6	7.66667	23799	23799	23799	78859	0.001±0.000	68988	18249	83369	92329	0.053±0.012
				3.8227	35.638	49.483	102.84		90.468	0.1472	13.561	164.66	
199	Propanoic acid, 2-phenylethyl ester	122-70-3	36.6667	63153	23246	22873	04177	0.001±0.000	0923	23799	24952	66974	0.081±0.021
				0.1472	169.47	139.41	670.35		67.795	0.1472	915.63	0.1472	
200	Propylene Glycol	57-55-6	40.5833	23799	38908	5802	64961	0.225±0.141	8327	23799	06208	23799	0±0
				0.1472	0.1472	47.718	68.327		129.47	243.98	107.53	77.317	
201	R-(-)-1,2-propanediol	4254-14-2	44.9167	23799	23799	11115	77006	0.834±0.329	701	8283	91135	62136	0.600±0.268
				2.0652	1.8733	1.1949	0.1472		5.7931	38.518	32.696	100.84	
202	Styrene	100-42-5	18.8333	70177	57911	07648	23799	0.001±0.000	27562	67927	4353	18285	0.101±0.055
				163.14	92.091	174.01	71.363		58.207	1997.1	118.40	91.627	
203	Terpinen-4-ol	562-74-3	28.25	53346	67491	26323	09625	0.009±0.004	48916	93743	13334	22591	0±0

				72.419	0.1472	0.1472	0.1472		55.190	60.600	276.31	108.78	
204	Tetraethylene glycol	112-60-7	47.25	24559	23799	23799	23799	0.964±0.255	90108	82784	03324	84096	12.370±3.425
		30361-34-		90.715	77.760	121.11	0.1472		20.690	11.863	40.337	10.770	
205	trans, trans-Octa-2,4-dienyl acetate	3	28.4167	99196	96593	72253	23799	0.001±0.000	48314	79816	39051	78049	0.254±0.199
				0.1472	0.1472	0.1472	300.44		509.42	603.48	575.32	555.32	
206	trans-β-Ocimene	3779-61-1	11.25	23799	23799	23799	225	0.609±0.153	03466	79631	72675	20264	2.594±0.892
				3.1846	2.3552	3.6116	140.13		38.530	54.531	216.30	181.24	
207	trans-Carveol	1197-07-5	33.5	42477	55567	8006	65707	0.001±0.000	1805	65102	70897	6286	20.813±5.934
				885.82	736.21	779.10	1096.9		1751.3	1154.9	1595.2	1246.8	
208	Trichloroacetic acid, 3-tridecyl ester	0-00-0	23.25	54852	49363	03387	57006	0.019±0.003	7895	39028	26751	32159	1.334±0.232
				28.536	0.9060	0.1472	1471.3		2934.1	3465.8	4217.0	3595.7	
209	Trichloromethane	67-66-3	11	725	46701	23799	40586	0.076±0.082	17756	67422	7105	34364	0.358±0.183
				14.924	0.1472	0.1472	135.31		74.455	0.1472	80.832	6011.9	
210	Triethylene glycol	112-27-6	40.4167	6396	23799	23799	31599	0.001±0.000	43494	23799	2778	5086	0.153±0.044
				0.1472	29.457	201.40	38.330		88.034	108.20	7.8176	0.1472	
211	Triethylene glycol monomethyl ether	112-35-6	45.1667	23799	06549	31657	57819	0.564±0.642	16146	84172	75373	23799	0.330±0.129
				107.02	83.822	109.38	0.1472		0.1472	0.1472	141.66	16.183	
212	Undecanal, 2-methyl-	110-41-8	18.8333	97043	10896	63389	23799	0.629±0.077	23799	23799	58355	25281	0.127±0.014

Table S2 Regression equation and linearity range (μg/mL n=8)

	Regression equation	Linearity range (μg/mL)	Related Coefficientt
hydroxy-α-sanshool	y=7.8504x+19.582	20~1000	0.9993
hydroxy-β-sanshool	y=4.4179x+3.3323	20~1000	0.9998
hydroxy-γ-sanshool	y=3.917x+30.392	20~1000	0.9987

Table S3 Degree of precision (n=6)

	Peak area						RSD value(%)
hydroxy-α-sanshool	4054.5	4054.5	4232.1	4071.4	4091.7	4083.8	1.6448
hydroxy-β-sanshool	4590.8	4590.8	4593.2	4602.9	4623.4	4625.9	0.353
hydroxy-γ-sanshool	8150.2	8150.2	8131.5	8181.6	8201.8	8198	0.3543

Table S4 Research on the reproducibility

	Sample content (μg/mL)					RSD value(%)
hydroxy-α-sanshool	12.63926	13.60939	13.83916	13.25198	12.25632	5.0434
hydroxy-β-sanshool	26.09106	25.29883	29.03364	25.59308	24.52923	6.6261
hydroxy-γ-sanshool	729.5193	716.4499	801.9869	788.357	859.1305	7.4284

Table S5 Research on the stability

	Injection time/h						RSD/%
	0	2	4	6	8	10	
hydroxy-α-sanshool	75.6	87	78.7	75.1	88.1	85.7	7.24
hydroxy-β-sanshool	120.1	121.8	115.4	119	137.8	131.6	6.89
hydroxy-γ-sanshool	5588.5	6073	5561.5	6187.3	6223.6	5669.4	5.27

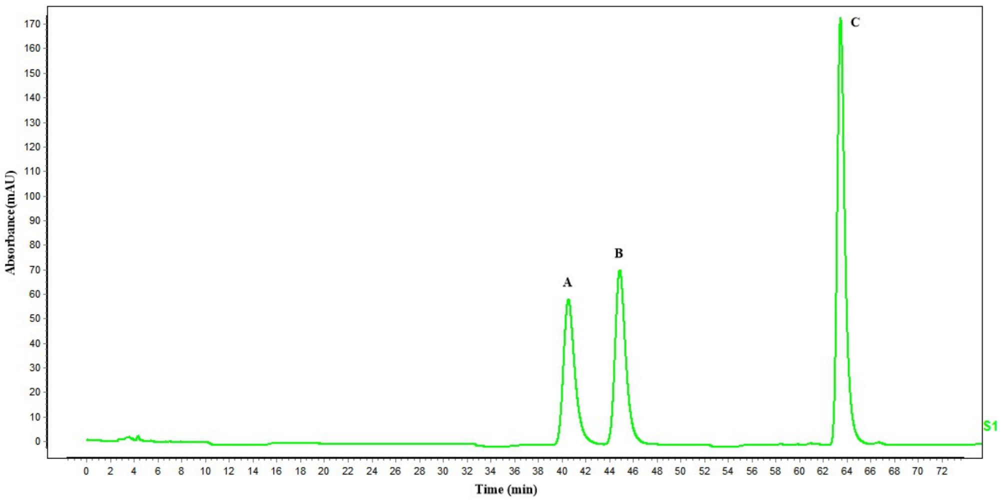

Figure S1 HPLC chromatogram of mixed standard of three compounds. A: hydroxy-α-sanshool; B: hydroxy-β-sanshool C: hydroxy-γ-sanshool

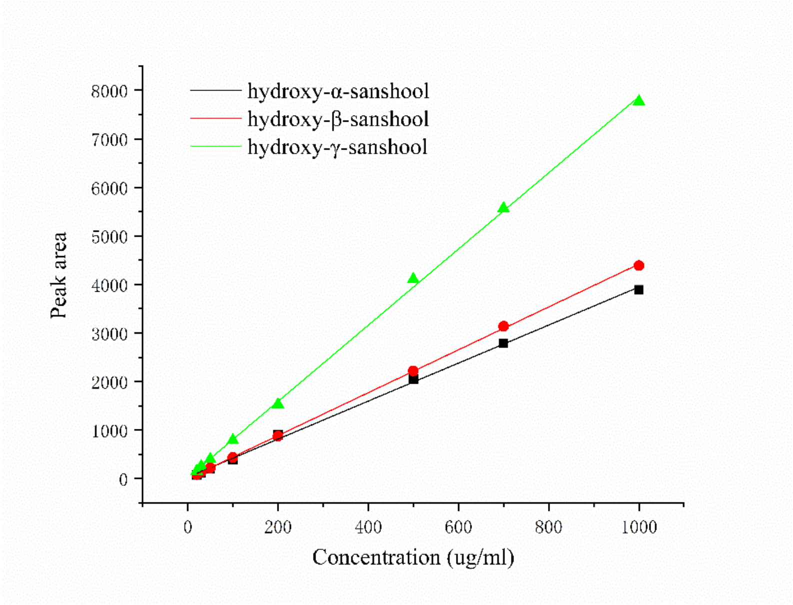

Figure S2 Standard curve of three sanshools
